# Comparisons of Citizen Science Data-Gathering Approaches to Evaluate Urban Butterfly Diversity

**DOI:** 10.3390/insects9040186

**Published:** 2018-12-06

**Authors:** Kathleen L. Prudic, Jeffrey C. Oliver, Brian V. Brown, Elizabeth C. Long

**Affiliations:** 1 Entomology, University of Arizona, Tucson, AZ 85721, USA; 2 School of Natural Resources and the Environment, University of Arizona, Tucson, AZ 85721, USA; 3 Office of Digital Innovation and Stewardship, University of Arizona, Tucson, AZ 85721, USA; jcoliver@email.arizona.edu; 4 Department of Entomology, Natural History Museum of Los Angeles County, Los Angeles, CA 90007, USA; bbrown@nhm.org; 5 Daniel Smiley Research Center, Mohonk Preserve, New York, NY 12561, USA; 6 La Kretz Center for California Conservation Science, University of California Los Angeles, Los Angeles 90095, CA, USA

**Keywords:** BioSCAN, California, iNaturalist, Lepidoptera, Los Angeles, Malaise trap, Pollard walk

## Abstract

By 2030, ten percent of earth’s landmass will be occupied by cities. Urban environments can be home to many plants and animals, but surveying and estimating biodiversity in these spaces is complicated by a heterogeneous built environment where access and landscaping are highly variable due to human activity. Citizen science approaches may be the best way to assess urban biodiversity, but little is known about their relative effectiveness and efficiency. Here, we compare three techniques for acquiring data on butterfly (Lepidoptera: Rhopalocera) species richness: trained volunteer Pollard walks, Malaise trapping with expert identification, and crowd-sourced iNaturalist observations. A total of 30 butterfly species were observed; 27 (90%) were recorded by Pollard walk observers, 18 (60%) were found in Malaise traps, and 22 (73%) were reported by iNaturalist observers. Pollard walks reported the highest butterfly species richness, followed by iNaturalist and then Malaise traps during the four-month time period. Pollard walks also had significantly higher species diversity than Malaise traps.

## 1. Introduction

Successful conservation relies on species occurrence data and their analysis. Research clarifies where a species is found, how and when it reproduces, and what aspects of its environment are important to its survival. All of this information contributes to a conservation framework from which policy is built. Research by professionals, be it in government or the academic realm, often requires intensive, unsustainable effort. Costly resources must be allocated across time and space, thus limiting the number of species for which this information is gathered, and ultimately, the number of species or habitats protected. Furthermore, the biological and physical systems of our planet are undergoing rapid changes as the impacts of human activity become ubiquitous. Obtaining biodiversity data quickly, effectively, and broadly has never been more important for conservation efforts. Urban areas can be species-rich habitats, especially for insects, but they are also a challenging environment in which to document biodiversity. Sampling and experimental designs are complicated by a heterogeneous built environment where access and landscaping are highly variable due to human activity [1,2]. It is projected that 10% of earth’s landmass will be urbanized by 2030 [3], important [4]. Citizen science projects may provide a valuable approach in urban areas [5]. Indeed, most citizen science surveys often occur within two hours of travel of an urban center due to the travel behaviors of volunteer scientists [6,7]. Citizen science focused on biodiversity is a broad descriptor with many data collection approaches, yet little is known about the tradeoffs among survey methods, especially for insects, and how effective and efficient they are in urban areas.

Citizen science, the collection and contribution of scientific data by the general public, is emerging as a method to collect large amounts of biodiversity data quickly, especially within urban areas. This approach has been shown to expand biodiversity research taxonomically, geographically, and temporally by building on a tradition of volunteerism and public engagement [8,9,10]. In the United States, citizen science has a long tradition of acquiring scientific-grade data. These community-contributed data are being increasingly used by scientific professionals in a variety of domains (e.g., [11,12,13,14]). This surge in the utility of citizen science-collected data is due largely to the development of web platforms such as eBird (www.ebird.org [6]), eButterfly (www.e-butterfly.org [15]), and iNaturalist (www.inaturalist.org [16]) that curate and host the data at broad geographic scales. However, more specialized citizen science projects are also valuable for specific taxonomic groups, leading to new discoveries. For example, the Natural History Museum of Los Angeles’ BioSCAN project discovered 43 new species of phorid flies in the Los Angeles basin [17,18], as well as highly unexpected drosophilid flies and parasitoid wasps [19,20].

Butterfly research has a long history of engaging the public in data collection, especially in the field of taxonomy [15,21]. Butterflies are comparatively easy to collect, handle, identify, and photograph. Because of this, butterflies are used as a bioindicator for other pollinator species and as an umbrella species for the protection of other insects and their host plants (e.g., [22,23]). Butterfly richness and diversity can be an indicator for the impacts of climate change and conservation reintroduction policy and procedures (e.g., [24,25]). Scarce data exists to compare citizen scientist efforts, professional surveys, and grassroots efforts by the public working on butterflies, thus leaving a gap in how to sample insect richness and diversity in urban areas. 

Here, we report on the relative merits of three popular citizen science approaches to assess butterfly richness and diversity in the Los Angeles Basin: organized, active surveys (ButterflySCAN Pollard walks) conducted by trained volunteers; organized, passive traps (BioSCAN Malaise traps) operated by trained volunteers and sorted by taxonomic experts; and incidental crowd-sourced observations (iNaturalist observations) vetted by the citizen science community. We discuss the tradeoffs of each approach in terms of resources and outcomes, providing guideposts for butterfly surveys and their role in urban conservation and development. 

## 2. Methods

### 2.1. Sampling Design

**Figure 1:**
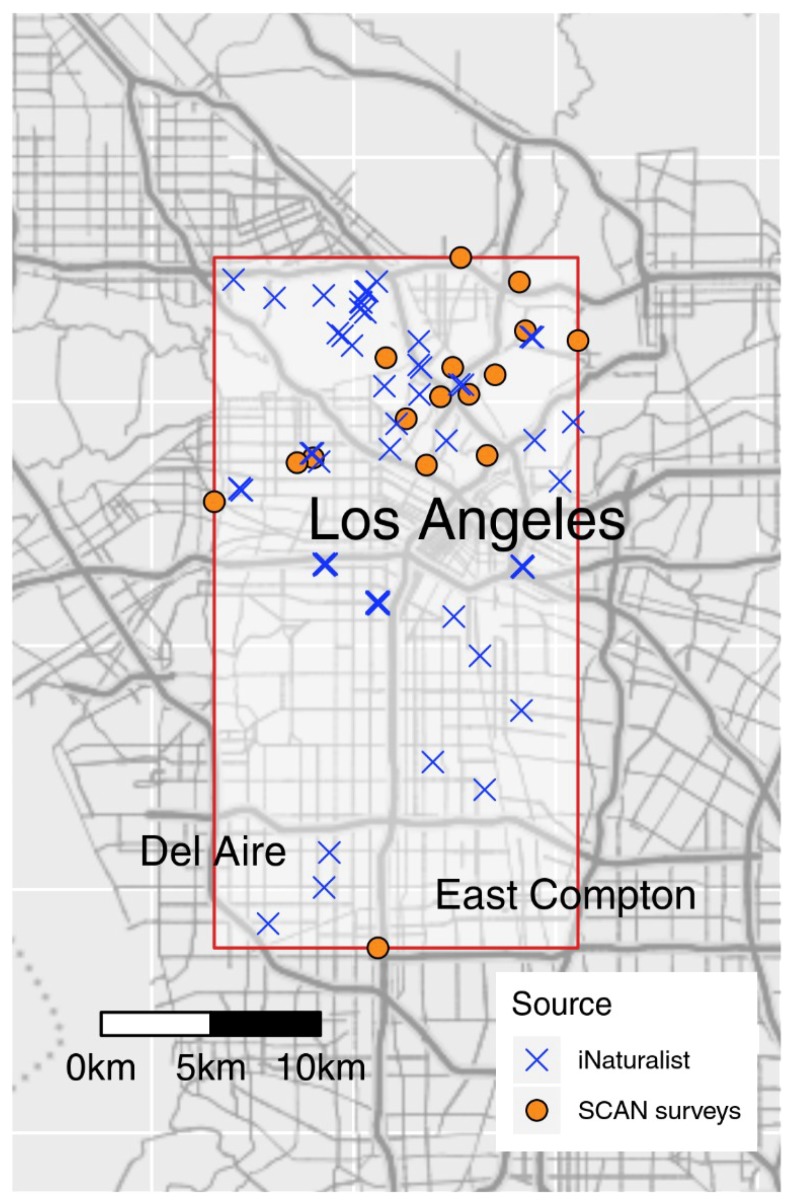
Sites included in this study. Orange circles indicate sites from the ButterflySCAN and BioSCAN approaches, while blue crosses show the sites of iNaturalist observations used in this work. The red rectangle defines the coordinate bounding box from which iNaturalist observations were sampled.

This study was carried out in the city of Los Angeles, California, USA, as part of the Natural History Museum of Los Angeles’ (NHMLA) BioSCAN and ButterflySCAN projects [18,26] (Figure 1). Of the 30 total BioSCAN sites available, 16 were selected for both Malaise traps and Pollard walks based on coverage continuity. All sites were located on private property and surveyed with the owners’ permission. Sites included homes, a school, and a nature garden (more site details are given in [26]). Sites were surveyed over a four-month period, between 15 March 2015 and 15 July 2015.

### 2.2. BioSCAN Malaise Traps

Malaise traps [27] were placed at 30 sites around Los Angeles and collected continuously for over one year as part of the NHMLA BioSCAN project. Samples used in this study were collected continuously between 15 March 2015 and 15 July 2015. Insects captured by the traps were funneled into a collecting bottle filled with 95% ethanol. Sample bottles were replaced every two weeks. Collected samples were butterflies were identified to the species level. The data given here reflect the pooled species abundance for each site. 

### 2.3. ButterflySCAN Pollard walks

Modified Pollard walks were conducted in the vicinity of each sampling site between 15 March 2015 and 15 July 2015 [28] by trained volunteers as part of the NHMLA ButterflySCAN project. Training included 3–5 guided walks with a butterfly expert, followed by verification of species identification via photographs. Sampling sites correspond to those used for Malaise trap sampling. Surveys were conducted by independent observers at each site. The Malaise trap sampling site was used as the starting point for each Pollard walk route. The observer then walked a route corresponding to a 1-km diameter circle around the start point over a 1-h period using public sidewalks and roadways. Species counts were made on the basis of visual observation. The observer recorded each species that was encountered during the survey and uploaded the data to the e-Butterfly web platform to share and archive the data (www.e-butterfly.org [15]). The data here reflect the pooled species abundance for each site. 

### 2.4. Citizen Science ButterflySCAN Pollard walk Training

NHMLA recruited over 20 participants to survey butterflies through Pollard walks as part of their ButterflySCAN citizen science program. Each participant was assigned to survey one sampling site via the modified Pollard walk protocol described above. Participants received 6 h of in-person, hands-on group training with experts to identify butterflies as well as individualized training at their survey site. Participants were also given butterfly identification guides specific to the butterfly fauna of Los Angeles. For the first two months, observers took photo documentation of each species recorded in order for project staff to verify the observers’ species determination. Only sites that had six or more surveys were included in this study. 

### 2.5. iNaturalist Data

To compare ButterflySCAN surveys and BioSCAN traps to an independent citizen science effort, we downloaded comparable iNaturalist data from the Global Biodiversity Information Facility (GBIF, [29]). Briefly, we downloaded all research-grade iNaturalist observations (over 10 million observations from all taxa [30]). We then restricted the observations to include only observations of butterflies observed between 15 March 2015 and 15 July 2015. We next restricted the data to only include observations within an area defined by the 16 BioSCAN and ButterflySCAN sites included in the study (Figure 1), resulting in 105 iNaturalist observations. We used the taxonomy of Pohl et al. [31] and reconciled differences between BioSCAN/ButterflySCAN and iNaturalist taxonomies (*Poanes melane* Edwards is known as *Paratrytone melane* in iNaturalist). Additional reconciliation was necessary due to differences in taxonomy between iNaturalist and GBIF: records of *Papilio rumiko* Shiraiwa and Grishin from iNaturalist are imported by GBIF and (mis)classified as *Zerynthia rumina* Linnaeus. We excluded one record of *Atlides halesus* Cramer from the iNaturalist data set because the geographic uncertainty was over 2500 km, making it an unreliable observation for comparing to BioSCAN and ButterflySCAN data, resulting in 104 iNaturalist observations being used in the current work. 

### 2.6. Statistical Analyses

We compared total species richness and species diversity between the two SCAN survey methods (Pollard walk and Malaise trap) with a paired *t*-test, assuming equal variance. For species diversity, we used Shannon’s *H* [32], which accounts for abundances in diversity calculations. Given the differences between the dedicated sampling of SCAN data (controlled survey time and area) and the incidental sampling of iNaturalist observations, we could not apply standard comparisons of means. To estimate the mean species richness of the geographical area included in the BioSCAN and ButterflySCAN projects, we calculated the total species richness for all sites for each of the three sampling approaches (Pollard walk, Malaise trap, and iNaturalist observations) between 15 March 2015 and 15 July 2015. To estimate uncertainty in the mean values, we performed bootstrap sampling with replacement to generate a distribution of mean total species richness. For the BioSCAN and ButterflySCAN data, this was done using a site/sampling method combination as the unit of resampling, while iNaturalist data sampled individual observations. All bootstrap samples replicated the observed number of sampling “events” (*n* = 16 for SCAN data, *n* = 104 for iNaturalist data). We performed *t*-tests on 1000 bootstrap samples to assess significant differences in total species richness between iNaturalist and the remaining two survey methods. While sampling “events” are not themselves comparable between the three survey methods, especially between the two SCAN surveys and iNaturalist observations, they represent the units that could be resampled in bootstrap estimates to compare species richness across the entire geographic area encompassing the SCAN sites. All data and source code is available in Supplementary Materials.

## 3. Results

**Figure 2:**
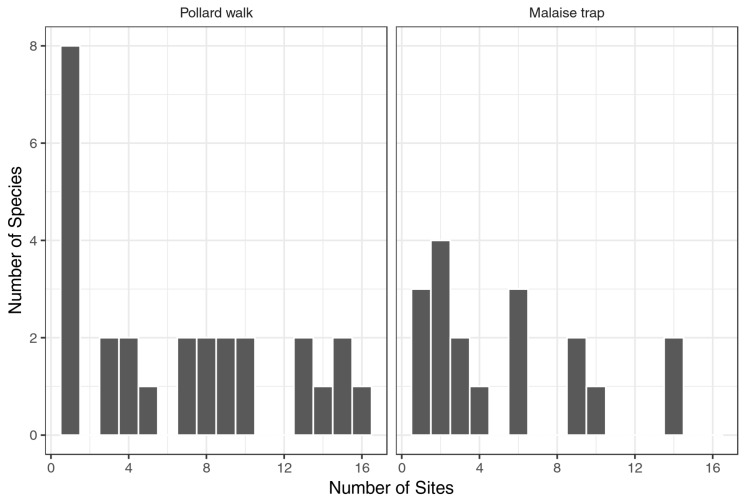
Frequency distribution of individual species in ButterflySCAN and BioSCAN surveys. Plots show the number of sites that each species was observed at in Pollard walk and Malaise trap surveys.

In the ButterflySCAN and BioSCAN surveys, a total of 28 species were recorded during the sampling period (Table 1). Both Pollard walks (27 species total) and Malaise traps (18 species total) included common species (those found at several different sites) and rarer species (those found at one or a few sites) (Figure 2). Notably, eight of the 27 species recorded during Pollard walks were observed at only one site.

**Figure 3:**
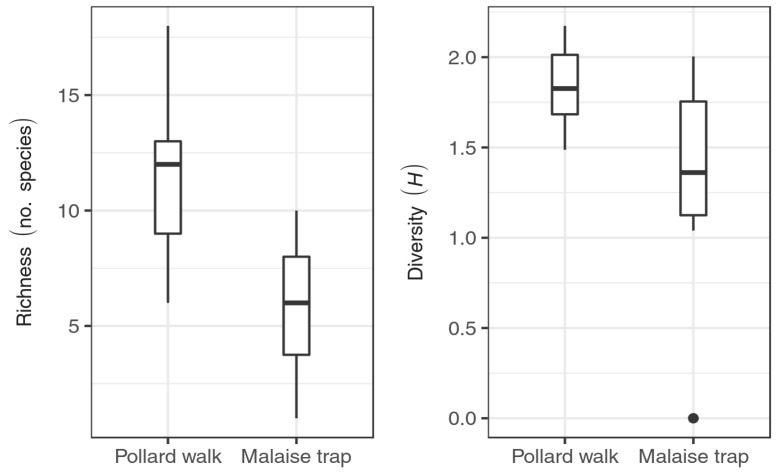
Species richness and diversity (Shannon’s *H*) observed across 16 BioSCAN Malaise traps and 16 ButterflySCAN Pollard walk sites between 15 March 2015 and 15 July 2015. Central lines are median values, boxes bound the first and third quartiles, and whiskers indicate 1.5 times the interquartile range (IQR).

Pollard walks had both higher species richness and higher diversity than did Malaise traps (Figure 3). Mean species richness was 11.31 species in Pollard walks and 5.94 species in Malaise traps. In pairwise comparisons between the two survey types, Pollard walks had, on average, 5.38 more species than Malaise traps (*t* = 4.66, *p* < 0.001). Shannon’s *H* was also higher in Pollard walks (*H* = 1.85) than in Malaise traps (*H* = 1.39) (*t* = 3.31, *p* = 0.005).

The total species richness in iNaturalist observations for the same area and time span was intermediate between Pollard walks and Malaise traps. The 104 iNaturalist observations included 22 species, including two (*Adelpha californica* Butler and *Limenitis lorquini* Boisduval) not recorded in either Pollard walks or Malaise traps. In the bootstrap resampling of observations, the differences among the survey types were significant, with Pollard walks reporting higher species richness for the area than iNaturalist (1000 pseudoreplicates, *t* = 66.80, *p* < 0.001) and Malaise traps reporting lower species richness for the area than iNaturalist (1000 pseudoreplicates, *t* = 62.65, *p* < 0.001). 

## 4. Discussion

One of the primary obstacles confronting conservation science and intervention is a lack of detailed species data. Obtaining such data can be costly and operate with a time lag that hinders positive conservation outcomes. Urban areas further complicate data collection because of a heterogeneous built environment where access and landscaping are highly variable due to human activity negatively influencing sampling design. Citizen science approaches may reduce the financial and time costs associated with species richness and diversity data collection and be particularly productive in an urban environment where volunteers frequently venture. The ButterflySCAN Pollard walks, where volunteers were assigned specific sites and interacted with a taxonomic expert, reported the most butterfly species (Table 1). iNaturalist, the community-driven, opportunistic citizen science approach with more variable expert interaction reported 22 butterfly species, and BioSCAN surveys, with passive Malaise traps located in volunteers’ backyards, reported 18 butterfly species (Table 1). ButterflySCAN Pollard walks also detected more rare species than Malaise traps (Figure 2). Malaise traps have been shown to be the least effective way of sampling Lepidoptera in more undeveloped environs [33]. iNaturalist data had some taxonomy glitches. Taxonomic differences and data sharing between iNaturalist and GBIF required a fair amount of taxonomic expertise to achieve accurate untangling. For example, records of *Papilio rumiko* Shiraiwa and Grishin from iNaturalist were imported by GBIF and (mis)classified as *Zerynthia rumina* Linnaeus. The latter species is native to the Iberian Peninsula, not the Los Angeles Basin, leading to the possibility of misinterpreting this record as a potential species introduction.

Species richness is the most basic and widely used measure of biodiversity [34]. However, it is an elusive quantity to measure properly [35]. ButterflySCAN Pollard walks provided higher estimates for species richness compared to iNaturalist and BioSCAN Malaise traps in the urbanized Los Angeles Basin. Observed species richness is strongly influenced by sampling effort [36], with individual based approaches such as Pollard walks and iNaturalist being better for a lower number of surveys (<100) than sample-based approaches such as Malaise traps [36]. Thus, our observed results confirm that even with volunteers collecting data, Pollard walks and iNatualist are a better approach for estimating richness than Malaise traps sorted by a taxonomic expert. iNaturalist data are stored “by observation”, not by survey, and thus calculation of Shannon’s *H*, our measure of diversity, was not possible.

Many studies suggest the power and potential of citizen science to provide data for conservation efforts, especially in a quickly changing environment (e.g., [8,9,10]). However, citizen science is a broad description of data-gathering approaches with varying degrees of engagement between scientists and volunteers. Here, we compared three approaches within the citizen science framework. For butterflies, Pollard walks still produced the highest species richness and abundance. This approach is more labor-intensive and requires the most coordination by the investigator. Single observations driven by the iNaturalist community produced better results than Malaise traps, although the data quality needs to be carefully considered. Sampling and reporting bias remains fully undescribed between the two citizen science methods. The Pollard walks here used a presence—absence approach in the protocol, while the iNaturalist observations record presence-only data. How these two sampling protocols interact with citizen science psychology and motivation is a future area of study (e.g., are more “photogenic” species reported on iNaturalist than using other survey methods?). Furthermore, we suspect that expert-driven engagement with volunteers also increased the detection of butterfly species, although our design did not separate training from the citizen science approach. This interpretation is in line with other evaluations of citizen science studies [7,15]. 

Moving forward, citizen science will continue to provide valuable scientific data for conservation efforts. Deciding on which citizen science approach to use will depend on the question and taxa under investigation, with an eye on data quality. For example, Pollard walks for a study on phorid flies is not a fruitful method, while Malaise traps are. Citizen science is most useful when considering the scale and scope of biodiversity surveys. Data collection can be scaled up to cover previously underexplored locations, as in the case of many urban environments. However, scientists will still need to engage with the volunteers to ensure sufficiently high data quality and make connections between volunteer actions and conservation results. Technological advances, such as machine-learning algorithms and chatbots, can be used in data-quality improvements, allowing scientific experts to reach more volunteers, helping them to learn more about the natural world, to produce better data, and to reach critical players in environmental conservation.

## 5. Conclusions

The future is bright for studying biodiversity in an urban matrix. Remote-sensing technologies and volunteered geographic information [37] are improving the spatial resolution and inference of habitat attributes. These data, linked with citizen science biodiversity data, can be pivotal for investigating urban butterfly ecology and conservation. Pollard walks are still the gold standard for obtaining valuable species richness data, especially when volunteers receive formal training from taxonomic experts. Recording these data on a community citizen science web platform such as e-Butterfly, which records data for species richness in its data-gathering protocol, is a great option for sharing data with the community, and it requires program management by engaging with the citizens to report their data. Citizen science is a powerful approach for conservation science, particularly in urban areas where many humans can act as sensors for biodiversity. 

## Figures and Tables

**Table 1:** Species observed in three data sources: ButterflySCAN Pollard walks, BioSCAN Malaise traps, and iNaturalist observations. For Pollard walks and Malaise traps, the number is the total number of sites where the species was observed; for iNaturalist data, the number is the total number of observations of species within the temporal and geographical limits of SCAN surveys (see Section 2: Methods).

Family	Species	Pollard walk	Malaise Trap	iNaturalist
Hesperiidae	*Erynnis funeralis*	9	6	4
	*Heliopetes ericetorum*	1	2	0
	*Hylephila phyleus*	13	9	17
	*Lerodea eufala*	10	9	4
	*Ochlodes sylvanoides*	1	0	0
	*Poanes melane*	4	14	4
	*Pyrgus albescens*	1	0	0
Papilionidae	*Papilio eurymedon*	1	0	0
	*Papilio rumjko*	3	1	2
	*Papilio rutulus*	10	0	4
	*Papilio zelicaon*	7	1	1
Pieridae	*Colias eurytheme*	8	0	0
	*Nathalis iole*	1	0	0
	*Phoebis sennae*	14	2	3
	*Pieris rapae*	16	14	4
	*Pontia protodice*	1	2	1
Nymphalidae	*Adelpha californica*	0	0	4
	*Agraulis vanillae*	15	6	9
	*Danaus gilippus*	1	0	0
	*Danaus plexippus*	15	4	15
	*Junonia coenia*	1	0	2
	*Limenitis lorquini*	0	0	1
	*Nymphalis antiopa*	5	0	4
	*Vanessa annabella*	4	2	2
	*Vanessa atalanta*	8	3	7
	*Vanessa cardui*	13	3	3
Lycaenidae	*Brephidium exilis*	0	1	2
	*Icaricia acmon*	3	0	0
	*Leptotes marina*	9	10	8
	*Strymon melinus*	7	6	3

## Supplementary Materials

Data from BioSCAN and ButterflySCAN surveys used in this work, along with all source codes for data processing and analysis, are available on GitHub at https://github.com/jcoliver/bioscan. Additionally, data and source codes are also archived at Zenodo (https://doi.org/10.5281/zenodo.1436741).
